# The Significance of Physiological Spaces in the Body and Its Medical Implications

**DOI:** 10.34133/2020/7989512

**Published:** 2020-03-04

**Authors:** Kezhen Zhang

**Affiliations:** Beijing Taijitang TCM Hospital, Haidian, Beijing 100097, China

## Abstract

The human body is made up of physical structures and spaces. These physiological spaces play an integral role in metabolic processes. In previous scientific research, these spaces were often neglected. The study of the importance of their existence in the body can help scientifically understand some of the thorniest medical problems that we currently face. It may also help in the diagnosis and prevention of disease, especially of drug-induced origins. The proper reading of the essence of meridians through the perspective of these spaces can help to improve scientific understanding of the principles of acupuncture and moxibustion and the treatment principles of Chinese and Western medicine and ultimately promote the communication and integration of Chinese and Western medicine.

## 1. Introduction

The research on basic theory and practical methods of life sciences is based on the composition of the human body. The correct understanding of the human body structure is the premise for studying the laws of life and the laws of disease. The main goal of our previous research is on visible, quantifiable tissues, organs, cells, or molecules that have specific morphological structures and ignores the spaces that are also present in the human body. As of now, there is no detailed report on its research [1]. This paper explores its impact on the body's metabolism and its role in diagnosis and treatment and prevention of diseases from the perspective of physiological spaces. In addition, a new understanding of physiological spaces can help to solve a series of difficult problems in medical theory, such as finding out the real cause of many diseases with unknown etiology, how the disease progresses from unformed to formed, how to detect and prevent diseases at an early stage, and how to reduce the formation of iatrogenic and drug-induced diseases.

This article also utilizes the special research perspective of physiological space and considers and examines the aspects of philosophy, medical basic theory, clinical, etc., providing a new research angle for many difficult problems in traditional Chinese medicine, such as meridians. What is the essence of meridians? Why are the answers so varied after so many years of research? Research has found that “meridians are relatively stable and relatively orderly spaces in the human body” [1]; this conclusion is consistent with the traditional Chinese medicine (TCM) theory, compatible with modern medicine, and can be confirmed by modern scientific experiments and clinical practice.

Traditional Chinese and Western medicine are two different medical systems that have been debated for more than a century. When studying and interpreting the principles of Chinese and Western medicine through the perspective of physiological spaces, the TCM theory resembles modern logic in many ways, making it easier to understand and accept. At the same time, this perspective may also help to resolve some of the disputes that exist between Chinese and Western medicine.

In summary, after we incorporate the spaces in the human body into our research horizon, the basic theory of medicine, the diagnosis and treatment of diseases, and the treatment methods would be improved and more practical. There is also a higher chance that many nagging medical problems may be solved.

## 2. The Human Body Is Made Up of Spaces and Physical Structures

### 2.1. Physiological Spaces Need to Be Incorporated as Part of the Foundation in Medical Research Theory

The phenomenon of space is something humans have experienced throughout history [2].

Democritus believed that the origins of all things are atoms and the void. An atom is a particle of matter that cannot be further subdivided, and a void is the place where the atom moves [2]. Therefore, the emptiness of the early atomists (kenon) should be understood as the gap between two atoms (diastema) [2].

But a human's experience of space is complicated and diverse. First, to say that anything exists, it must mean that it is located somewhere. Something that does not exist in any place does not exist. This is the experience of position, location, and place. Second, people all know that there is a state of “emptiness.” For example, after a meeting has ended and people have dispersed, the space in the room is the experience of a “void.” Third, people know that objects differ in size and shape and have different length, width, and height. This is the so-called extension experience [2]. In classic Greek natural philosophy, the experience of the space, the experience of the void, and the extended experience have not been integrated into a unified space concept [2].

Therefore, “the experience of space has different types. If you need to find the root of the space problem, you must understand the various relationships of the space and unify it in a concept of ‘space.' Describing and analyzing how humans experience the characteristics of spaces has long been one of the most attractive and important tasks in philosophy” [3].

The modern philosopher John Philoponus integrated three spatial experiences, arguing that space is “purely extended, providing space for objects but can also be separated from objects when in thought, so that there is no real presence of void” [2].

These three types of experiences of space are also a new understanding of the objective world from the perspective of space. Spatial experience is an understanding of the location of the object; extension experience is the understanding of the size and shape of the object; and the experience of the void is a reconsideration of the state and relationship of the object, which will vary depending on different academic backgrounds and experiences. From this perspective, many of the previous understandings of space are one-sided. For example, when a child of modern times has not been exposed to knowledge of physics or chemistry, he would say that “water” is continuous and does not know that “water” is composed of molecules. When we ask him if there is something in the room besides what is visible, he will either say nothing or that it is filled with continuous “air” [4].

Therefore, space exists everywhere, regardless of the fact that if it is solid, liquid, or gaseous substances, they are all filled with spaces. Even molecules or atoms can be further divided and are filled with space. But space is not “nothing.” It is just that human beings have been living in a space full of gaseous substances since birth. They are used to treating it as “existing” space or “empty,” just as fish will ignore water. In fact, within what we think is “empty,” there are still substances that are not visible to us.

But there is a problem: the “space” that different people understand or recognize is not completely consistent or even uncertain. How should we understand “space”? Is there a standard? The ancient Chinese philosophers solved this problem ingeniously through contrast. Lao Tzu believed that “when there is no comparison, it is difficult to form a relationship, to measure against each other….” [5]. This kind of comparative point of view, with the different manifestations of various substances, gives us the difference between “space” and “solid entity” in our actual experience. If there is a glass of water in the room, we will think that water and cups exist as solid entities in the air-filled space, and if there is a stone in the water, we will think that the stone as a solid exists in the water-filled space.

This unique Chinese experience of space can be intuitively felt and creatively applied in real life. For example, the modern philosopher Heidegger was influenced by the “existence and emptiness” concepts in Chapter 11 of Lao Tzu, and he used the kettle as a metaphor to illustrate the role of space. A pot exists as a container. When we fill the pot, we find that the vessel is able to contain something. The void is what allows the item to contain something [6].

The famous architect Frank Lloyd Wright was also influenced by the idea of Chapter 11 of Lao Tzu and applied creatively in architecture. He even engraved the words of Lao Tzu on the wall of the Taliesin West to show their importance: “The reality of the building does not Consist in roof and walls but in the space within to be lived in. - Lao Tzu” [7].

Renewed understanding of space is of great significance for us to understand the laws of life and the patterns of disease. The human body is composed of space and solid entity. Solid and space are a pair of opposite and interdependent existences. They exist relative to each other as a reference [1]. If one party disappears, the other party loses the premise for existence. When one party changes, the other party will inevitably change accordingly. In the process of studying the laws of the human body, it would obviously be considered wrong to ignore the solid entity, but if the space is neglected, this would also be a mistake. Therefore, from the philosophical level, the neglect of physiological spaces will inevitably lead to serious defects in the basic theory of medicine.

### 2.2. The Omnipresence of Space in the Body

Previously, in the understanding of the construction of the human body, we generally focus on the physical structures that can be dissected, which are made of tangible substances and measurable, such as organs, tissues, cells, and molecules. But a similarly important part was completely ignored: the physiological spaces that also exist in the body.

From the perspective of a human body structure, space exists widely in the human body, such as the nasal cavity, ear canal, oral cavity, intestinal tract, and lung, which are filled with the gases and communicate with the natural space outside the human body. These spaces play an important role in normal metabolism and function. Specific to the various parts of the human body, the internal structure of the organ is also inseparable from the participation of space. As in the lungs, the trachea, bronchi, and alveoli are made up of space. For example, in the circulatory system, we can regard the blood vessel wall as a kind of solid entity, and the intravascular channel and the flowing blood can be regarded as a relative space; compared with the blood cells in the blood, the plasma is a space, and blood cells can be seen as an entity in the plasma.

In the human body, between organs, tissues, and cells, there exists a multitude of spaces that take different forms.

Even an internal structure commonly thought of as solid is filled with spaces. Any organs or tissues are made up of molecules, which in turn are made up of atoms. If you blow up an atom to the size of a basketball court, the nucleus is smaller than the size of a ping-pong ball. The nucleus can still be further split into protons and neutrons, which means that there is still space inside the nucleus. By dividing this way, you can see more clearly the fact that space is everywhere, and smaller entities can be broken down into more microscopic spaces step by step.

Once we take physiological spaces into account, then we need to seriously rethink and reposition our various understandings of life sciences and medical sciences. Not only do we need to rethink the composition of the human body, but also it will be crucial to understanding disease, diagnosis, prevention, etiology, the interpretation of traditional medicine, the improvement of the existing medical theory system, and the diagnosis and treatment system.

## 3. Physiological Spaces Are a Precondition for Normal Metabolic Processes in the Body

**Figure 1:**
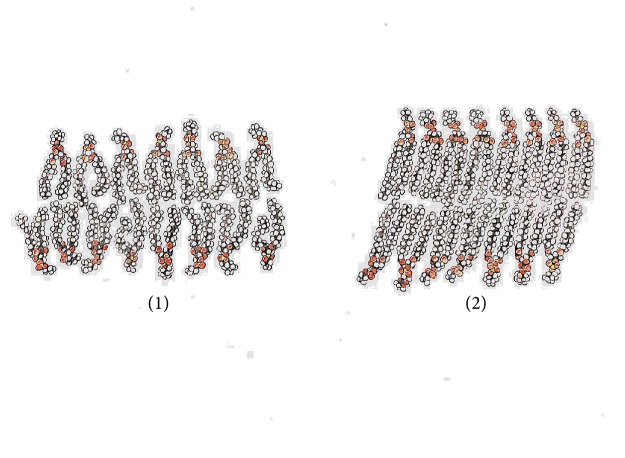
Schematic of spaces inside the cell membrane [8]. (1) Under normal temperature and (2) after lowering of temperature.

1. Normal physiological spaces are where the functions of the human body and its organs, tissues, cells, etc. take place. The spaces inside the lungs must exist for gas entry and blood oxygen exchange to occur. In the lungs of someone with lobar pneumonia, liquid exudation occupies the spaces inside the normal lung, leading to gas being unable to enter the alveoli, and therefore irregularities in the process of gas exchange. In patients having an asthma attack, spasms cause the narrowing of the bronchi, which prevents the free flow of air, and various symptoms occur as a result
2. Normal physiological spaces also act as the channels for the exchange of internal materials and energy supply. The energy supply of the human body cannot be separated from the normal blood circulation. Normal blood flow provides nutrients to cells in every corner of the body. If the spaces in the blood vessels are compressed or blocked, the nutrient channels will be blocked. The same is true at the microscopic level. For example, the exchange of energy between cells is provided by the space in the cell membrane lipid bilayer with permeability (see Figure 1). If the spaces in the cell membrane shrink, normal metabolism of the cell will inevitably be affected
3. Physiological spaces are the channels through which metabolic waste is discharged. For example, in the digestive system and the urinary system, the waste form after food is digested and absorbed needs to be excreted through the intestines and the urinary tract
4. Physiological space is also a channel for information transmission. In the nervous system, whether the conduction of information is normal is closely related to space. For example, a synaptic structure, which is a key part of the functional connections between neurons, plays an important role in the process of nerve conduction and is also a key part of information transmission. During this process, whether or not the synaptic cleft directly is normal affects the normal release and transmission of synaptic vesicles in the presynaptic membrane
5. Normal physiological spaces are also channels for communication and coordination between the inside of the human body and the external environment. The skin is a barrier separating the inside and outside of the human body. The spaces inside the skin play an important role in the internal/external exchange and the regulation of the human body. For example, the heat generated by the metabolism of cells in the human body will be released to the outside of the body through the normal spaces in the skin, which maintains the normal temperature of the human body. If the spaces inside the skin shrink due to cold temperatures and other factors, the channels for heat dissipation will be impacted and body temperature will become abnormal

In conclusion, almost all the functions within the human body require the participation of spaces. Once these physiological spaces become abnormal, disease will result.

## 4. Taking a Second Look at the Significance of Physiological Spaces in Disease Diagnosis, Treatment, Prevention, and Disease Etiology

### 4.1. Understanding Physiological Spaces inside the Body Will Help Early Detection and Effective Treatment of Diseases

In clinical practice, inertial thinking has led us to treat and prevent diseases focused on the idea of the body as a solid entity, for example, the morphological changes of organ tissues, the changes of various laboratory tests, and the number and type of pathogens. The direct consequence of this type of diagnosis and treatment is that it is only when the solid entity exhibits obvious abnormalities will it be detected by laboratory instruments and a diagnosis can be confirmed. Especially for some diseases that only exhibit changes in symptoms but the test results are normal, doctors often do not know how to interpret or how to treat them. This situation changes once we incorporate space into our understanding of disease.

Through the patterns of physiological spaces, diseases can be diagnosed more intuitively. The distribution of physiological spaces has a certain regularity. If the internal organ tissue changes, an abnormal reaction occurs in the corresponding body surface through physiological spaces. This is also the principle behind judging visceral diseases through body surface changes called diagnosis through observation and palpation in TCM clinical practice.

**Figure 2:**
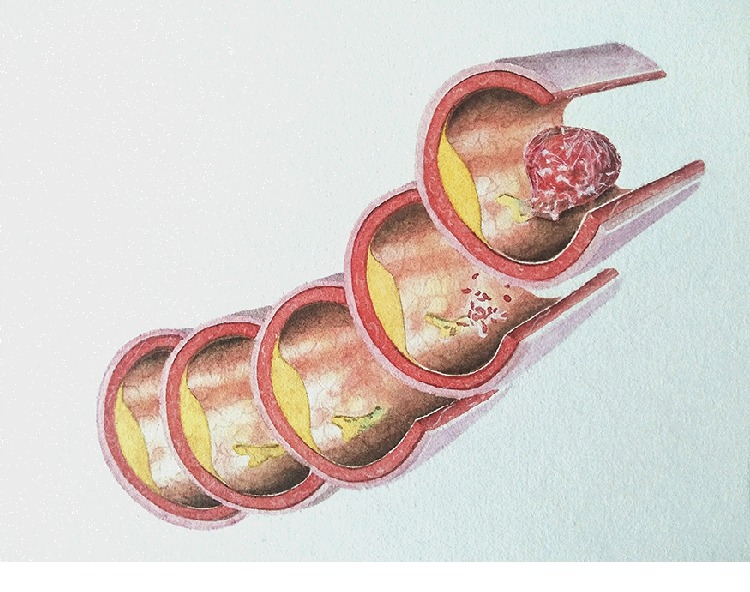
Schematic of thrombosis [8].

**Figure 3:**
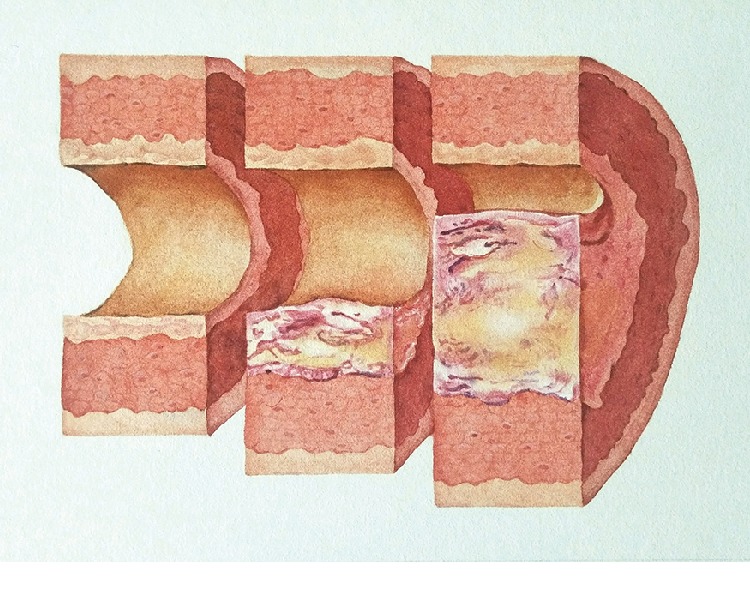
Schematic of atherosclerosis [8].

Through the changes in physiological spaces, early diagnosis and even prevention of diseases can be achieved. For many diseases, the process of going from mild to severe is also a process during which normal space is gradually occupied by entities. Examples are tumor formation, thrombosis, and formation of atherosclerosis (see Figures 2 and 3). If we understand the laws of space, we will be able to detect abnormal changes in space and intervene effectively before the formation of organic diseases, to prevent further disease development and even prevent disease.

### 4.2. Understanding Physiological Spaces Will Help Guide the Treatment of Diseases

The principle behind the treatment of many diseases is to restore their normal physiological spaces. If the urinary or bile duct stones are eliminated, the space is restored and the symptoms are relieved; the elimination of inflammation causes the exudation of the interstitial spaces to be absorbed, and the normal spaces in the tissue are restored; after the thrombus is ablated, spaces in the vascular channel are restored and the blood flow becomes smooth again; after the tumor is resected, the spaces in the tissue are restored, and the compression is relieved. Whether it is resectioning abnormal tissues or recovery of normal cellular metabolism, these all involve the change of spaces.

### 4.3. Once Physiological Spaces Are Understood, There Would Be More Treatment Methods and Ways to Apply Medicine

Currently, common routes for medication application are mostly through intravenous infusion or oral administration. Once we recognize the patterns of physiological spaces, there can be more ways to treat diseases. For example, we can treat visceral diseases through the surface of the skin and the spaces in the subcutaneous tissues or treat visceral diseases with acupuncture at the distal extremities. Changes in approach to treatment, especially rethinking the use of medication, will make clinical treatment more direct, simpler, more effective, and safer.

### 4.4. With Knowledge of Physiological Spaces, the Understanding of Etiology Can Also Be Repositioned

Previously, how we think about the cause of diseases is focused on mostly physical elements that are very specific, quantifiable, and tangible. Once physiological spaces are taken into consideration, the perception of the cause of diseases will also change completely.

Physiological spaces play a vital role in the body's metabolic processes; therefore, any factor that affects the physiological spaces may become a cause of disease. We all know that after a sudden drop in temperature, the incidence of disease will suddenly increase. However, the most common natural factors such as coldness are difficult to test with the current standards we use to detect the cause of disease. But from the perspective of physiological spaces, its pathogenic effect is obvious (see Figure 3). For example, in the neck tissues, blood vessels and nerves pass through the space of the muscles. When the cold temperature stimulates the neck, soft tissues such as the skin and muscles will contract, and the spaces become narrow. The blood vessels and nerves that pass through the neck then will correspondingly be compressed. Furthermore, this affects the normal channels of nutrient delivery and waste discharge, so the corresponding functions and metabolic indicators will also be affected.

Similarly, lifestyle and work habits, psychological factors, and so on will also become the cause of illness due to how they affect the normal physiological spaces.

Therefore, recognizing the cause of diseases from the perspective of physiological space is of great significance for the diagnosis, treatment, and prevention of diseases.

### 4.5. Understanding Physiological Spaces Help Us Further Understand the Operating Patterns of Life

Life is an enormous open system that is immensely complicated [9].

In the normal metabolic process of life, it is not enough that each individual component is operating normally. The interrelationship between them and the coordination in the process of life and metabolism are also important prerequisites for maintaining normal life.

Different entities have different organizational differences, and the direct relationship between them is affected by the structure of the organization and has certain limitations. But the spatial differences between different entities are much smaller; they can connect the whole body and even the inside and outside of the body as a complete whole.

Physiological space is ubiquitous in the human body. Apart from providing a place for the existence and operation of various parts of the body, as well as providing energy supply and waste discharge channels for the body's metabolism, these ubiquitous spaces also play a decisive role in the synergy between the various components of the metabolic processes in a living organism.

Recognizing this role that physiological spaces play will be of great significance for us to attain a deeper understanding of the metabolic patterns of life and the formation, diagnosis, and prevention of diseases.

## 5. A Whole New Understanding of Physiological Spaces and Chinese Meridians

“That which is called the twelve meridians are the sources from whence humans originate, disease form, humans are treated, and disease is borne” [10].

“The meridians can determine life or death, treat hundreds of diseases, modulate excess and deficiency, and hence must be kept open” [10].

Because the meridians are so important, they are considered the core and soul of the system of traditional Chinese medicine. But what exactly are meridians? This is a point that has confounded the medical world for ages.

### 5.1. Traditional Chinese Medicine Classics Have Explored the Characteristics of Space from Many Angles

In the Chinese medical classic, *Huang Di Nei Jing*, it is said that “the twelve meridians are connected to the *zang* and *fu* organs within, and are spread like a net through the limbs without” [10]. This implies that a person's five *zang* and six *fu* organs, as well as his limbs and his musculoskeletal system, are all connected into an organic, integrated whole by the meridians, which also coordinate the relative balance and order within the body so that all the functions in the body be appropriately performed. Therefore, meridians are the pathways through which the various organ and tissue systems in the body coordinate and communicate.

In the *Huang Di Nei Jing*, there are many more discussions regarding the pathway-like characteristics of the meridians.

- “Allow the Qi and Blood to flow and the Yin and Yang to be nourished” [10]: the meridians are the passageways through which the energy that enables metabolic processes within the body to occur is delivered
- “Reduce the excess, tonify the deficient, allowing the Yin and Yang to be restored” [10]: The meridians are the pathways through which diseases may be treated
- “When an external evil attacks the body, it must first adhere itself to the skin and hair; if it is not expelled, it will invade the tertiary collaterals. If it is still not expelled, it will advance into the meridians, which connect to the five *zang* organs, and spread itself among the stomach and intestines…” [11]: The meridians are also the channels through which disease and pathological changes are transmitted
- “The twelve meridians are hidden and roam within crevices of the flesh” [10]: This directly refers to how some of the meridians are the spaces within soft tissues such as muscles

In traditional Chinese medical meridian theory, the meaning of “points” actually means “a space,” which is also called “node” [12] or “cavitation” [12] and so on in the *Huang Di Nei Jing.* Some classical texts specifically emphasized the “space-like” characteristic of points and differentiated the points from physical structures such as the skin, muscles, tendons, and bones. Some texts stated “that which is referred to as a ‘node' is where the *shen* and Qi roams, entering and exiting the body, and not skin, flesh, tendons or bones” [10]. *Zhen Jiu Jia Yi Jing* (the A-B Canon of Acupuncture) used the word “cavity” directly when referring to “points” [13].

### 5.2. During Clinical Practice Using Acupuncture, the Space-Like Characteristic of Meridians Can Also Be Proven

When practicing acupuncture, an experienced acupuncturist can feel directly with his hand the condition of the space within the meridians. This is referred to in the ancient classics as “where the space for the acupuncture point is located is where the needle should be placed” [14] and “when needling here, one would most certainly hit the space of the acupuncture point and not the flesh joints; once the needle reaches the space in the acupuncture point, the sensation will follow the appropriate port onward” [10]. During the Tang Dynasty in China, the famous acupuncturist Shangshan Yang proposed that “the word ‘port' (*gang*, 港) used here means ‘the empty space [within the point]'” [15].

### 5.3. Modern Science Experiments Prove the Space-Like Characteristic of Meridians

In early 1950, when Dr. Yoshio Nakatani of Japan, Dr. J.E.H. Niboyet of France, and their contemporary researchers passed direct current through patients' skin, they demonstrated that the electricity conductivity was higher in areas that fit the classical description of the meridians [16]. In their analysis of the causes for the occurrence of high power or low resistance, using biological knowledge and theories that were already widely accepted, Dr. Weisheng Yang et al. deduced that “the reason for low resistance in the meridians was because of the relatively higher content of interstitial fluids (tissue fluids)” [17]. The reason that the meridians contain a higher degree of tissue fluid is precisely due to the existence of space, allowing the different substances in the body to be able to seep into and fill the area and thereby changing the conductivity. But whenever there is a disease condition, the structure of the space within the meridians will also change. Therefore, during an experiment, there may be times where the conclusion is drawn that “the low resistance feature of the points will change due to the existence of disease.”

In 1987, at the Chinese Academy of Acupuncture, Dr. Jingbi Meng and his associates used radioisotope to conduct a study on ten meridians, including the Pericardium channel, the San Jiao channel, the Stomach channel, the Liver channel, and the Gall Bladder channel. The movement of the isotope was steady and clearly in a linear fashion. When the tracks left by these movements were traced, they were mostly in line with the meridians as described by ancient Chinese medical texts [18]. The route and speed of the movement, as well as the fact that the movement is easily affected by externally applied pressure and environmental factors such as low temperature, are all characteristics that point to the theory that the meridians are actually the spaces that exist in the body. They also indicate that meridians are not, as some theories have proposed, known body structures such as nerves or blood vessels.

### 5.4. From How the Human Body Is Constructed, One Can Also See That the Characteristics of Physiological Spaces and Meridians Match

In the human body, regardless of its hair, organs, soft tissue, or hard bones, between the tissues and between the cells, going as miniscule as between the molecules, space is everywhere. It is precisely this space that links all the different tissues and cells in the body into one connected, integrated, and cooperative whole. Combining both the description of the meridians in classical Chinese medical texts and conclusions from modern scientific experiments, no other existing and known systems match the meridians more so than the spaces that are present throughout the body. Rather, only the spaces in the body fit all the characteristics that meridians possess.

Even though large sums of evidence can be found through both scientific experimentation and clinical practice, why cannot people find or see the actual structure of the meridians during autopsies? This is because of the space-like characteristic of the meridians. Because their structure is actually just made up of space and the space is located between the other physical structures of the body, once the area is cut open during an autopsy, the physical structures around the space would be destroyed instantly, which means the empty space will also be changed or even disappear completely. This is the same idea as when a cup has been smashed to bits; the space wherein water may fill up also disappears along with the physical structure of the cup. But emptiness does not signify the lack of function; rather, this space is an objective existence that has a function that is directly related to its emptiness. The same reasoning applies to the meridians in the body.

Research has found that “meridians are relatively stable and relatively orderly spaces in the human body” [1]; this conclusion is consistent with traditional Chinese medicine theory and compatible with modern medicine and can be confirmed by modern scientific experiments and clinical practice.

## 6. Physiological Spaces and the Principles of Chinese and Western Medicine

### 6.1. Physiological Spaces Need to Be Incorporated as Part of the Foundation in Medical Research Theory

Traditional Chinese medicine is a life science with a unique theoretical system and model for clinical thought. Its foundational work, The Yellow Emperor's Internal Classic, was formed as early as the Warring States Period more than 2,000 years ago. In fact, the contents of certain chapters are from much earlier times and may even be traced back to the Shang Dynasty (about 1600-1046 BC) [19].

Despite thousands of years of medical practice experience and a mature disease diagnosis and prevention model, it has been difficult to make TCM compatible with modern medicine, and it is difficult to interpret it and apply it within modern scientific language and systems. However, from the new perspective of the research into the physiological spaces, TCM diagnosis and treatment ideas that used to be difficult to understand are no longer contradictory to the logic of Western medicine but are just presented as a different perspective. This also unintentionally sets up a bridge of conciliation between Chinese and Western medical communication.

**Figure 4:**
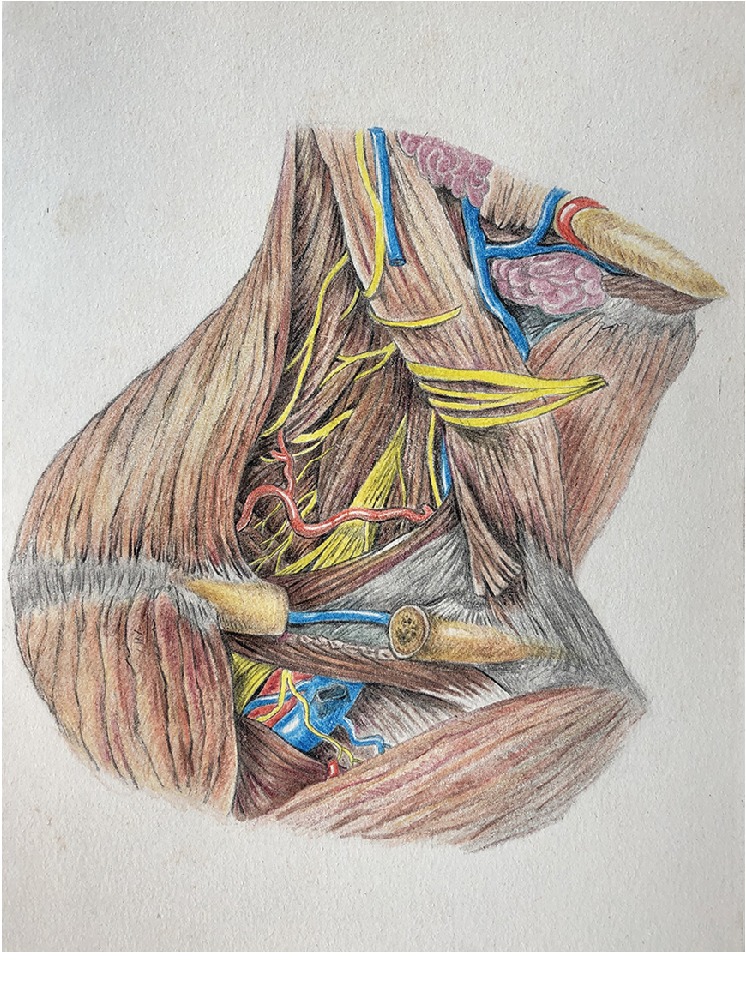
Blood vessels and nerves passing through the spaces within the muscles [8].

**Figure 5:**
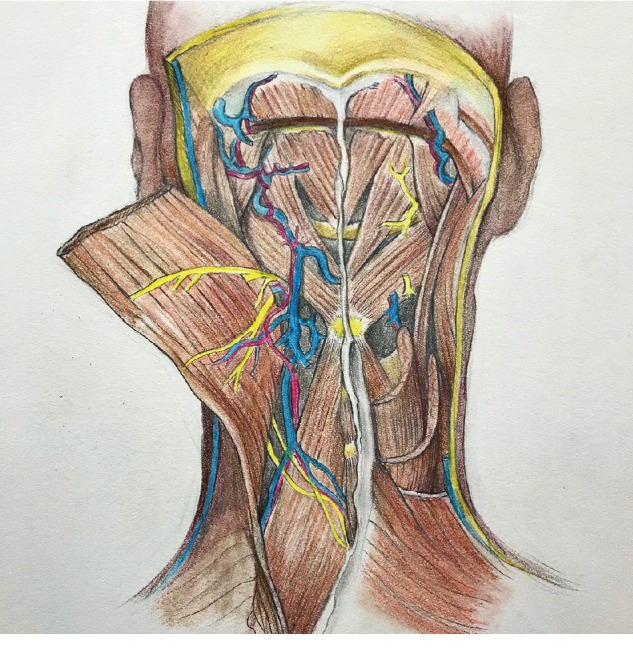
Blood vessels and nerves passing through the spaces within the muscles [8].

Take for example the common cold. Modern medicine usually thinks that a cold is caused by bacteria or viruses [20], but the experience “catching a cold” tells us that most colds usually occur after a sudden drop in temperature or the body was exposed to cold. The words “catching a cold” are even closely related to “cold”? Why is catching a cold related to “cold”? Why is there a series of symptoms such as fever, cough, and tonsil swelling after catching a cold? From the perspective of anatomical structures, the blood vessels and nerves that reach the head, pharynx, larynx, trachea, etc. pass through the gap between the neck muscles (see Figures 4 and 5). After being stimulated by the cold, soft tissues such as muscles would contract due to the stimulation by the cold. When contraction occurs, the interstitial space becomes relatively narrow, and the blood vessels and nerves that pass through are subjected to different degrees of compression, affecting the normal circulation of blood to the above-mentioned parts. The nervous system will inevitably be affected as well [1]. As a result, the patient may develop various symptoms depending on the affected area, such as headache, dizziness, sore throat or dry itching, stiff neck and back pain, tonsil enlargement, and coughing. During the time of treatment, Western medicine would perform the corresponding symptomatic treatment according to the different symptoms. The traditional Chinese medicine theory, on the other hand, believes that these phenomena are caused by “wind-cold seizing up the surfaces,” that is, a contraction state of the soft tissue on the body's surface after being triggered by the wind and cold.

**Figure 6:**
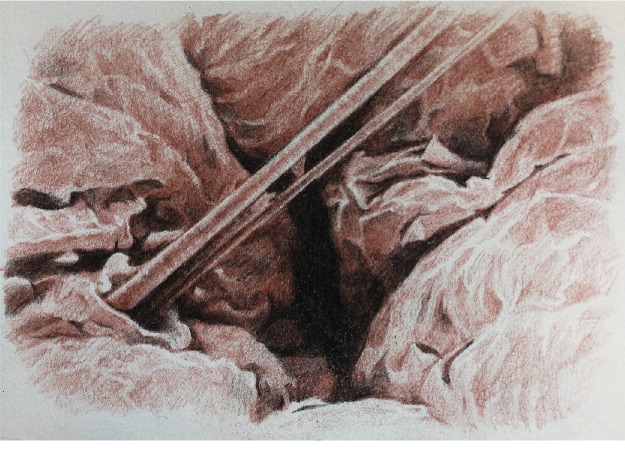
Skin (magnification 1000x) [8].

After catching a cold, a common symptom that follows is a fever. The traditional Western medicine theory believes that this symptom occurs due to dysfunction in the body's temperature regulation center. However, from the perspective of physiological spaces, under normal circumstances, the spaces that exist in the skin (see Figure 6) allow for heat generated by the metabolism of cells in the body to be released to the outside of the body, so that the body temperature regulation is maintained within the normal temperature range (such as an underarm temperature of 36.2-37.2 degrees Celsius). If the spaces under the skin shrink after catching a cold, the channel for external heat dissipation is also blocked, and the excessive accumulation of internal heat causes the body temperature to rise or even generate high heat, which is referred to in the *Huang Di Nei Jing* as “what is said to be heat disease, all can be considered a type of cold damage” [11].

For the treatment of such diseases, traditional Chinese medicine does not treat the heat source or the thermoregulatory center. Instead, it uses the method of “spicy warm releases exterior,” that is, using such formulas as *Mahuang Tang*, *Guizhi Tang*, *Guizhi Jia*, and *Gegen Tang*. The mechanism of action of these formulas is to (1) relax the spaces that became contracted due to the cold surface of the body, so that internal heat can be dissipated outwards and the body temperature can be reduced to normal, and (2) allow the spaces inside soft tissues (such as muscles) to be relieved and restored. The pressure on the blood vessels and nerves that pass through is then relieved as well, and various symptoms, such as congestion and pain, are alleviated in a short time. In fact, the sweating and relaxing of muscle and other soft tissues that occur after taking antipyretic and analgesic drugs used in Western medicine for these symptoms are also ways to restore the spaces under the body's surface.

Other treatments in Chinese medicine can also be reasonably interpreted from the perspective of physiological spaces. For example, the method of purging downward [21] is to cause excretion of the stool remaining in the colon and restore the patency of the intestinal spaces. The method of resolving stagnation [22] is to restore the spatial passage in the blood vessel, so that the stagnation in the blood vessel can be resolved and blood can circulate freely again.

### 6.2. Physiological Spaces Are the Bridge for Chinese and Western Medicine to Communicate

As a system of medicine, both Chinese medicine and Western medicine serve human health, but the dispute between Chinese and Western medicine in modern times has been plaguing academic circles. “The practice of comparative study and combined research between Chinese and Western medicine has made us see that the academic differences between Chinese and Western medicine are almost incompatible” [23]. As early as more than one hundred years ago, some people even proposed to abolish Chinese medicine, declaring that “until the day the old doctor is removed, the people's thinking will remain unchanged, and the new medical advancement will not happen” [23]. But the facts tell us that Chinese medicine is indeed effective in clinical practice and can even cure some difficult diseases that other medical systems cannot treat. Even some scholars who opposed Chinese medicine in the past recognized this. “The theory of Chinese medicine is too mysterious and unscientific. Everyone admits this. But the miracle of Chinese medicine is also true” [23].

If Chinese medicine is indeed miraculous, why are they “unscientific methods” at the same time? An important reason is that the theoretical basis that Chinese medicine is founded on has not been objectively recognized, especially the renewed understanding of the essence of meridians and the principles of Chinese medicine treatment.

Meridians are the soul and core of the Chinese medicine system, yet during autopsy, its physical structure cannot be observed. Researchers have many hypotheses, contradictions, and so on that are difficult to unify. This inevitably affects people's perception of Chinese medicine. If the meridian cannot be proven to be “real,” then the TCM diagnosis and treatment system established on it will inevitably be considered to be inconsistent with “scientific principles.” However, when we realize that the meridians are the space that exists in the human body, the Chinese medicine diagnosis and treatment system has a basis for dialogue with modern science; then many problems now have common theoretical support. Because the objective fact of the space existing in the human body is whether it is anatomy or more microscopic cytological level, or even molecular biology, it can be intuitively recognized by the naked eye or instrument. It is just that we usually just observe the solid entities, and this mindset limits our research perspective. This proves that the Chinese medicine system is not contradictory to modern medical or scientific principles, but the perspective of understanding is different. In fact, in theory and practice, Chinese medicine has its own unique advantages.

Therefore, restoring normal space is a key prerequisite for curing various diseases. The analysis and treatment of diseases from the perspective of physiological space are based solely on changes in the physical state of abnormal spatial forms in the human body. It is not directly targeting physical factors such as molecular structure, cellular receptors, organ parenchyma, bacteria, and viruses, and the treatment method is more intuitive, simple, and safe. It does not need to achieve therapeutic purposes at the expense of damage to the physical structure and avoids the destruction of the solid tissue by the use of drugs, which is of great significance for avoiding the occurrence of drug-induced diseases.

Recognizing the existence of physiological spaces can not only complement and improve the foundational theories of medicine but also improve the existing defects in clinical diagnosis, treatment, and prevention. At the same time, from the new perspective of physiological spaces, the traditional Chinese medicine theory and clinical practice are easily compatible with the context and principles of modern medicine and modern science. It provides a bridge of communication and integration between Chinese and Western medicine, allowing for improvement in the current medical system so that it could better benefit human health.

## 7. Conclusion and Outlook

Currently, the medical field research on physiological space is still nearly completely absent. The main reason is that the habitual way of thinking causes us to pay too much attention to the solid entities and ignore the existence of the spaces. This has caused a serious flaw in the current medical basic theory, making it difficult to find a real cause, effectively predict the occurrence and development of disease, and effectively cure diseases, leading to higher iatrogenic and drug-induced diseases. Treatments are also costly and inefficient.

Some international cutting-edge studies seem to be developing an interest with a “spatial” structure. Some scholars have realized the spatial structure of the human body and its role in the human body, such as the “interstitial tissue” or “fluid filled space” and its “buffering effect” [24]. However, this kind of recognition is still different from the true understanding of physiological spaces. It still does not break away from the solid entity-centered way of thinking, which makes it difficult to truly understand the whole picture. As I wrote in the “The Theory of Physiological Spaces,” published in 2006, I systematically summarized various forms of space existence [1] and role [1].

In summary, the following conclusions can be drawn:

1. The human body is composed of two parts: spaces and solid entities. However, in the past, the entity-centered way of thinking led us to ignore the spaces that exist in the human body. Physiological space plays a decisive role in the metabolic processes of life. New understanding and studies can make up for many shortcomings in the current medical system
2. The interpretation of the essence of meridians from a spatial perspective has been fully proven in practice. A scientific understanding of the foundations of Chinese medicine and acupuncture and the principles of Chinese and Western medicine can be supported by the theory of physiological spaces, which can effectively promote the communication and integration of Chinese and Western medicine
3. Factors that affect physiological spaces can also lead to disease. This new understanding will enable us to reevaluate the causes of diseases. In medicine, many diseases considered to have “unknown cause” will find their true roots. Clinical treatment ideas will also be transformed from the result-based (such as the symptoms, signs, and indicators of laboratory tests) into etiology-based, which can effectively avoid the occurrence of iatrogenic and drug-induced diseases

The reinterpretation and understanding of the human body through the perspective of physiological spaces require that we reexamine the start and development of human physiology and pathology. The specific mechanism needs to be further explored.

## Data Availability

All data needed to evaluate the conclusions in the paper are present in the paper. Additional data related to this paper may be requested from the author.
